# The endemic gastropod fauna of Lake Titicaca: correlation between molecular evolution and hydrographic history

**DOI:** 10.1002/ece3.280

**Published:** 2012-07

**Authors:** Oliver Kroll, Robert Hershler, Christian Albrecht, Edmundo M Terrazas, Roberto Apaza, Carmen Fuentealba, Christian Wolff, Thomas Wilke

**Affiliations:** 1 Department of Animal Ecology and Systematics, Justus Liebig University GiessenGermany; 2 National Museum of Natural History, Smithsonian InstitutionWashington, D.C.; 3 Facultad de Ciencias Biologicas, Universidad Nacional del AltiplanoPuno, Peru; 4 Instituto de Ecología, Universidad Mayor de San AndresLa Paz, Bolivia; 5 Departamento de Zoologia, Universidad de ConcepcionChile

**Keywords:** Altiplano, *Heleobia*, molecular clock, phylogeography, species flock

## Abstract

Lake Titicaca, situated in the Altiplano high plateau, is the only ancient lake in South America. This 2- to 3-My-old (where My is million years) water body has had a complex history that included at least five major hydrological phases during the Pleistocene. It is generally assumed that these physical events helped shape the evolutionary history of the lake's biota. Herein, we study an endemic species assemblage in Lake Titicaca, composed of members of the microgastropod genus *Heleobia*, to determine whether the lake has functioned as a reservoir of relic species or the site of local diversification, to evaluate congruence of the regional paleohydrology and the evolutionary history of this assemblage, and to assess whether the geographic distributions of endemic lineages are hierarchical. Our phylogenetic analyses indicate that the Titicaca/Altiplano *Heleobia* fauna (together with few extralimital taxa) forms a species flock. A molecular clock analysis suggests that the most recent common ancestor (MRCAs) of the Altiplano taxa evolved 0.53 (0.28–0.80) My ago and the MRCAs of the Altiplano taxa and their extralimital sister group 0.92 (0.46–1.52) My ago. The endemic species of Lake Titicaca are younger than the lake itself, implying primarily intralacustrine speciation. Moreover, the timing of evolutionary branching events and the ages of two precursors of Lake Titicaca, lakes Cabana and Ballivián, is congruent. Although Lake Titicaca appears to have been the principal site of speciation for the regional *Heleobia* fauna, the contemporary spatial patterns of endemism have been masked by immigration and/or emigration events of local riverine taxa, which we attribute to the unstable hydrographic history of the Altiplano. Thus, a hierarchical distribution of endemism is not evident, but instead there is a single genetic break between two regional clades. We also discuss our findings in relation to studies of other regional biota and suggest that salinity tolerance was the most likely limiting factor in the evolution of Altiplano species flocks.

## Introduction

**Figure 1:**
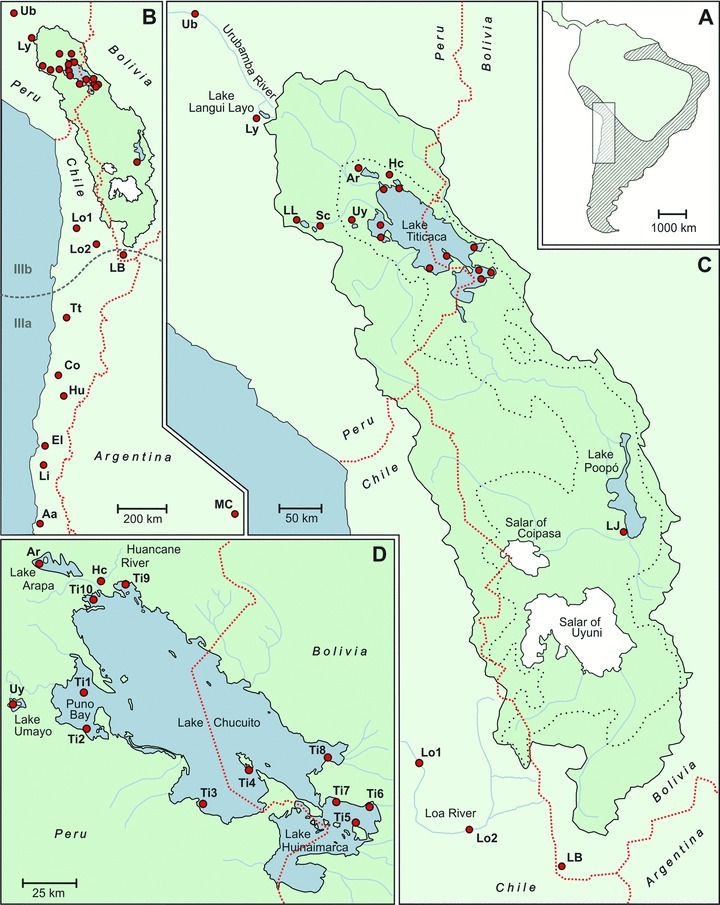
Sampling sites in the Altiplano (red circles). (A) Geographic range of the genus *Heleobia* (dashed area, modified from Hershler and Thompson 1992: map 6) and location of the sampling area (rectangle) in South America. (B) Closeup of the portion of (A) showing the sampling sites outside the Altiplano. The gray dashed line indicates the geographic distribution of the two main *Heleobia* clades IIIa and IIIb. (C) Closeup of the portion of (B) showing the sampling sites in the Altiplano and the major hydrologic features of the region. The maximum prior extant of Lake Titicaca is indicated by a dotted black line (Lavenú 1992; Wirrmann 1992). (D) Closeup of portion of (C) showing the sampling sites in Lake Titicaca and its two subbasins, Lake Chucuito and Lake Huinaimarca. For locality codes see Table 1.

Ancient Lake Titicaca (Peru/Bolivia), which is located in the northern part of the endorheic, high-elevation Altiplano (Dejoux 1994; Allmendinger et al. 1997; Pawley et al. 2001; Baker et al. 2005; Fig. 1), contains a diverse endemic fauna whose biogeographic history is poorly understood. The following two hypotheses have been proposed for the generation of endemic diversity in ancient lakes (e.g., Martens 1997). (1) These water bodies have functioned as sinks for extralimital biota over long time periods, resulting in the accumulation of phylogenetically diverse assemblages (reservoir function). (2) The lakes have served as a venue for local (intralacustrine) speciation (cradle function). Both of these contrasting scenarios assume that the paleohydrology of these lakes has played a pivotal role in the assembly of endemic biota (e.g., Martens 1997).

**Figure 2:**
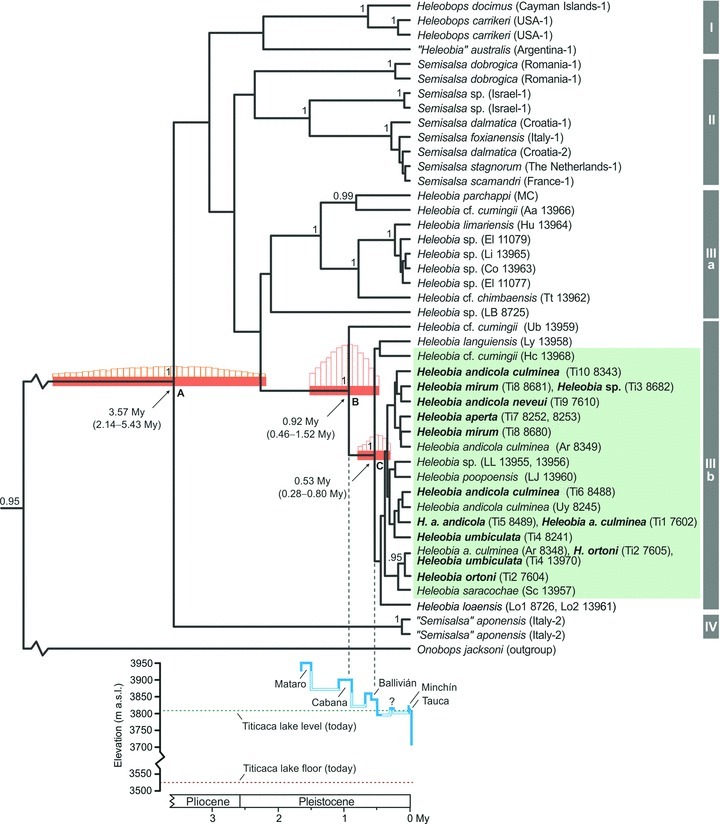
Bayesian phylogenetic tree under the strict clock model inferred from the mitochondrial COI gene (top). Two of the three outgroups (*Spurwinkia salsa*, *Cochliopa* sp.) were removed from the tree a posteriori. Specimens are labeled with a locality code according to Table 1. In addition, specimens of the genus *Heleobia* carry the respective DNA voucher number behind the locality code. Bayesian posterior probabilities are indicated when ≥0.95. Major clades are delineated by gray bars and labeled with Roman numerals. Lake Titicaca specimens are bold faced; specimens from the Altiplano are green shaded. The ages of MRCAs discussed in the text (labeled A–C) are provided and their 95% HPD intervals are illustrated by orange bars (associated node-depth distributions are indicated by white bars above the HPD intervals). Major paleohydrologic events in Lake Titicaca (including prior lake floor levels) are shown below (Early and Middle Pleistocene data are from Lavenú[1995]; Late Pleistocene and Holocene data are from Betancourt et al. [2000] and Blard et al. [2011]). The question mark refers to an unnamed prior configuration of Lake Titicaca (Lavenú 1995) that could be equivalent to the central Altiplano Escara period (Fornari et al. 2001).

The history of Lake Titicaca was punctuated by a series of major hydrologic events. The lake originated during the Late Pliocene/Early Pleistocene, about 2−3 million years (My) ago (Lavenú 1992) and underwent several phases of expansion and contraction during the Late Pleistocene that were caused by glacial–interglacial cycles and associated changes in effective moisture (Wirrmann 1992; Argollo and Mourguiart 2000; Cross et al. 2000; Fritz et al. 2007; Blard et al. 2011). At least five major phases have been recognized, which are sometimes referred to as “paleolakes” (Lavenú 1981, 1995; Lavenú et al. 1984; Wirrmann 1992; Cross et al. 2000; Baker et al. 2005; also see Figs. 1C and 2).

The Mataro Lake, coeval with the Purapurani formation (Calvario/Kaluyo interglacial S_5_) 1.5–1.6 My ago (Lavenú 1995; also see Lavenú et al. 1984; Marshall and Sempere 1991), was 3950 m in elevation (i.e., ca. 140 m higher than at present) and constituted the largest recorded expansion of Lake Titicaca, overlapping much of the Altiplano (Fig. 1C; Lavenú et al. 1984). Lake Mataro eventually desiccated and Lake Cabana, corresponding to the Kaluyo/Sorata interglacial (S_4_) (Lavenú 1995; also see Lavenú et al. 1984; Marshall and Sempere 1991), developed ca. 1.1–1.0 My ago with a maximum lake-level elevation of 3900 m. The third episode gave rise to Lake Ballivián with a lake level of 3860 m (Lavenú 1981; Lavenú et al. 1984). Most authors suggest that Lake Ballivián originated during the Sorata/Choqueyapu I interglacial (S_3_/t_3_) about 0.6–0.5 My ago (Lavenú 1995; also see Lavenú et al. 1984; Marshall and Sempere 1991; but see Fornari et al. 2001).

Subsequent climatic changes resulted in the North Minchin episode, corresponding to the Choqueyapu I/II (t_2_) interglacial (Lavenú 1995), approximately 73–30 kiloyears (ka) ago with a lake level of 3825 m (Fornari et al. 2001), and the North Tauca episode, corresponding to the postglacial Choqueyapu II (t_1_) phase (Lavenú 1995), approximately 18.0–14.5 ka ago with a lake level of 3815 m (Blard et al. 2011). Lake-level fluctuations continued into the Holocene; contractions of up to 100 m depth and drastically increased salinity levels have been reported for this time interval (Betancourt et al. 2000; Cross et al. 2000). It is generally assumed that these paleohydrologic events helped shape the evolutionary history of regional aquatic biota. For example, the repeated cycles of lake extension and shrinking may have promoted dispersal and vicariance, respectively; and desiccation and associated fluctuations in salinity may have resulted in extinction (e.g., Lüssen et al. 2003; Benavides 2005).

The Lake Titicaca region contains at least 533 aquatic species (Dejoux 1994); at least 64 of these (12%) are considered to be endemic (González and Watling 2003; Lüssen et al. 2003; Benavides 2005). However, these numbers are considerably smaller than in most other ancient lake basins (e.g., Martens 1997). The relatively small number of endemic species in this lake has been attributed to (1) the possibility that the ancestral biota was tropical in origin and consequently was depleted during the uplifting of the Altiplano because few species could tolerate high elevations and/or low temperatures (de Lattin 1967); and (2) the large variation in lacustrine water chemistry during the late Cenozoic, which resulted in extinctions (Wirrmann et al. 1991; Dejoux 1994).

Despite the relatively small number of endemic species in Lake Titicaca, there are possible species flocks of pupfishes (genus *Orestias*; e.g., Lüssen et al. 2003), amphipods (genus *Hyalella*; e.g., González and Watling 2003; Väinölä 2008), and microgastropods (genus *Heleobia*; e.g., Hershler and Thompson 1992). The phylogenetic relationships and biogeographic history of these three groups have not been well established, although the molecular evolution of *Orestias* has been detailed in an unpublished dissertation (Lüssen 2003). That study included preliminary molecular-clock analyses that suggest that speciation was recent and possibly associated with Middle to Late Pleistocene paleohydrologic processes.

Virtually nothing is known about the phylogenetic relationships of the *Heleobia* flock (14 species) in Lake Titicaca. Altiplano congeners are mostly endemic whereas extralimital members of the genus range more widely. This prevailing biogeographical pattern suggests that *Heleobia* may be a particularly suitable group for investigating evolutionary diversification in the Lake Titicaca region.

We here use a molecular clock approach together with a phylogeographical analysis to address the following questions:

1. Did Lake Titicaca serve as a biogeographic reservoir for Altiplano species or did the endemic snail lineages in the lake evolve through rapid intralacustrine speciation? This relates to the age and phylogenetic composition of the endemic fauna, and the extent to which evolutionary diversification occurred within the lake.
2. Are diversification events in *Heleobia* spp. related to major paleohydrological episodes on the Altiplano? The question is associated with processes of speciation in ancient Lake Titicaca and the abiotic factors driving evolution.
3. Are there hierarchical spatial levels of endemicity in the Lake Titicaca region? This question is related to the concept of ecological isolation of ancient lake species, that is, assumed low levels of faunal exchange between ancient lakes and their watersheds as well as between watersheds and extralimital areas.

This is the first phylogeographical study of an Altiplano invertebrate species assemblage and may contribute to a better understanding of speciation processes in Lake Titicaca. Furthermore, given that Lake Titicaca differs from most, if not all other ancient lakes in its physical and biotic features, this study may help identify the unifying patterns and processes in world-wide ancient lakes that, in general, can explain their often outstanding degree of biodiversity.

## Methods

### Study species and sampling sites

*Heleobia* Stimpson 1865 is one of three genera belonging to the primarily brackish water subfamily Semisalsinae Giusti and Pezzoli 1980 (Caenogastropoda: Rissooidea: Cochliopidae). The other two are *Semisalsa* Radoman 1974 (treated as a subgenus of *Heleobia* by some authors) and *Heleobops* Thompson 1968. This subfamily is composed of small (typically 2–8 mm in shell height), dioeceous fresh water and brackish water gastropods that usually live on hard substrates or aquatic vegetation.

*Heleobia* is distributed in South America from central Peru south to the Tierra del Fuego, along the eastern coasts of Argentina and Brazil, and in the Amazon Basin (Fig. 1A; also see Hershler and Thompson 1992). The center of diversity of the genus is the Altiplano, which contains 20 species, the majority of which are endemic to Lake Titicaca (Haas 1955, 1957; Blume 1958; Hershler and Thompson 1992).

Our study is based on hierarchical sampling that included (1) eight *Heleobia* taxa from Lake Titicaca, (2) six *Heleobia* taxa from other Altiplano areas, (3) representative *Heleobia* taxa from the Andes and the Chilean coastal region, (4) two congeners from Argentina, (5) seven members of the sister genus *Semisalsa* from Europe and western Asia, and (6) two species of the genus *Heleobops* from North and Central America (Table 1).

**Table 1:** Collection and locality data (latitude, longitude, and elevation in parentheses), locality code, DNA voucher number (UGSB collection), and GenBank accession numbers for specimens analyzed in this study. Information on outgroup species is given in the text. *Sequences from GenBank (references in parentheses), **sequences provided by Hsiu-Ping Liu (Metropolitan State College of Denver)

Taxon	Collection site	Locality code	DNA voucher number	GenBank accession number*
*Heleobops*				
*Heleobops docimus* Thompson 1968	Cayman Islands, Pond at Chisholm Point (19.3486°N, 81.2293°W, 0 m), leg., det.: R. Hershler	Cayman Islands-1	n/a	AF129322 (Hershler et al. 1999)
*Heleobops carrikeri*Davis and McKee 1989	USA, Oyster Pond, Falmouth (41.5345°N, 70.6395°W, 0 m), leg., det.: G. M. Davis	USA-1	584 597	JQ973018 JQ973019
*Semisalsa*				
*“Semisalsa” aponensis* (E. von Martens 1858)	Italy, thermal spring near Villaga, Vicenza (45.40°N, 11.53°E), leg., det.: I. Niero	Italy-2	3242 3245	JQ973020 JQ973021
*Semisalsa dalmatica* (Radoman 1974)	Croatia, Pirovac Spring, Pirovac (43.8167°N, 15.6766°E), leg., det.: A. Falniowski and M. Szarowska	Croatia-1	2114	AF367631 (Wilke et al. 2001)
	Croatia, Krka River, Skradin (43.8172°N, 15.9281°E, 7 m), leg., det.: A. Falniowski	Croatia-2	2099	JQ973022
*Semisalsa dobrogica* (Grossu and Negra 1989)	Romania, Movile Cave, Mangalia (43.825°N, 28.561°E), leg., det.: not specified in original publication	Romania-1	n/a n/a	EU938128 EU938132 (Falniowski et al. 2008)
*Semisalsa foxianensis* (De Stefani 1883)	Italy, thermal springs, Torretta establishment, Montecatini Terme (43.89°N, 10.77°E), leg., det.: S. Cianfanelli & E. Loro	Italy-1	3760	JQ973023
*Semisalsa stagnorum* (Gmelin 1791)	The Netherlands, Kaaskenswaters, Zierikzee (51.65582°N, 3.93580°E, 0 m), leg., det.: T. Wilke	The Netherlands-1	2915	JQ973024
*Semisalsa scamandri* (Boeters et al. 1977)	France, Étang du Charnier, Saint Gilles (43.62°N, 4.31°E, 0 m), leg., det.: H. Girardi	France-1	3088	JQ973025
*Semisalsa* sp.	Israel, Mouth of Nahal Taninim River, Ma’agan Michael (32.5386°N, 34.9029°E, 0 m), leg., det.: F. Ben-Ami & J. Heller	Israel-1	2005 2148	JQ973026 JQ973027
*Heleobia*				
*Heleobia andicola andicola* (D’Orbigny 1835)	Bolivia, Lake Titicaca, Patapatani Island (16.306°S, 68.686°W, 3809 m), leg., det.: O. Kroll	Ti5	8489	JQ973028
*Heleobia andicola culminea* (D’Orbigny 1840)	Peru, Lake Titicaca, Ramis Bay (15.324°S, 69.831°W, 3809 m), leg., det.: O. Kroll	Ti10	8343	JQ973029
	Bolivia, Lake Titicaca, Huarina (16.209°S, 68.621°W, 3809 m), leg., det.: O. Kroll	Ti6	8488	JQ973030
	Peru, Lake Titicaca, Puno Bay (15.453°S, 69.552°W, 3809 m), leg., det.: O. Kroll	Ti1	7602	JQ973031
	Peru, Lake Arapa, Arapa (15.147°S, 70.104°W, 3815 m), leg., det.: O. Kroll	Ar	8348 8349	JQ973032 JQ973033
	Peru, Lake Umayo, Umayo Island (15.739°S, 70.171°W, 3840 m), leg., det.: O. Kroll	Uy	8245	JQ973034
*Heleobia andicola neveui* (Bavay 1904)	Peru, Lake Titicaca, Vilque Chico (15.236°S, 69.695°W, 3809 m), leg., det.: O. Kroll	Ti9	7610	JQ973035
*Heleobia aperta* (Haas 1955)	Bolivia, Lake Titicaca, Chua (16.120°S, 68.449°W, 3809 m), leg., det.: O. Kroll	Ti7	8252 8253	JQ973036 JQ973037
*“Heleobia” australis* (D’Orbigny 1835)	Argentina, Mar Chiquita, Cangrejito inlet (37.7442°S, 57.4195°W, 69 m), leg., det.: R. Hershler (USNM 1002579)	Argentina-1	n/a**	JQ972708**
*Heleobia* cf. *chimbaensis* (Biese 1944)	Chile, Quebrada de Taltal (25.503°S, 70.411°W, 577 m), leg., det.: O. Kroll	Tt	13962	JQ973038
*Heleobia* cf. *cumingii* (D’Orbigny 1835)	Peru, Huancane River (15.216°S, 69.792°W, 3815 m), leg., det.: O. Kroll	Hc	13968	JQ973039
	Peru, Urubamba River, Urubamba (13.312°S, 72.110°W, 2861 m), leg., det.: O. Kroll	Ub	13959	JQ973040
	Chile, Aconcagua River, Concon (32.916°S, 71.497°W, 0 m), leg., det.: O. Kroll	Aa	13966	JQ973041
*Heleobia languiensis* (Haas 1955)	Peru, Lake Langui Layo (14.452°S, 71.280°W, 3999 m), leg., det.: O. Kroll	Ly	13958	JQ973042
*Heleobia limariensis* (Biese 1944)	Chile, Huasco River, Vallenar (28.579°S, 70.765°W, 380 m), leg., det.: O. Kroll	Hu	13964	JQ973043
*Heleobia loaensis* (Biese 1947)	Chile, Loa River, Quillagua (21.637°S, 69.549°W, 812 m), leg.: U. Bößneck, det.: O. Kroll	Lo1	8726	JQ973044
	Chile, Loa River, Calama (22.453°S, 68.903°W, 2260 m), leg., det.: O. Kroll	Lo2	13961	JQ97304
*Heleobia mirum* (Haas 1957)	Bolivia, Lake Titicaca, Ajilata (16.011°S, 68.819°W, 3809 m), leg., det.: O. Kroll	Ti8	8680 8681	JQ973046 JQ973047
*Heleobia ortoni* (Pilsbry 1924)	Peru, Lake Titicaca, Chucuito (15.882°S, 69.898°W, 3809 m), leg., det.: O. Kroll	Ti2	7604 7605	JQ973048 JQ973049
*Heleobia parchappi* (D’Orbigny 1835)	Argentina, Mar Chiquita, Canal Ea (37.5468°S, 57.3128°W, 69 m), leg., det.: R. Hershler (USNM 1002582)	MC	n/a**	JQ972709**
*Heleobia poopoensis* (Bavay 1904)	Bolivia, Laca Jahuira River (19.079°S, 67.314°W, 3696 m), leg., det.: O. Kroll	LJ	13960	JQ973050
*Heleobia saracochae* (Haas 1955)	Peru, Lake Saracocha (15.764°S, 70.621°W, 4154 m), leg., det.: O. Kroll	Sc	13957	JQ973051
*Heleobia umbiculata* (Haas 1955)	Bolivia, Lake Titicaca, Sol Island (16.044°S, 69.156°W, 3809 m), leg., det.: O. Kroll	Ti4	8241 13970	JQ973052 JQ973053
*Heleobia* sp.	Peru, Lake Titicaca, Chocasuyu (16.205°S, 69.398°W, 3809 m), leg., det.: O. Kroll	Ti3	8682	JQ973054
	Peru, Lake Lagunillas (15.706°S, 70.806°W, 4174 m), leg., det.: O. Kroll	LL	13955 13956	JQ973055 JQ973056
	Bolivia, Lake Blanca (22.812°S, 67.766°W, 4323 m), leg.: U. Bößneck, det.: O. Kroll	LB	8725	JQ973057
	Chile, Copiapo River, Atacama (27.808°S, 70.128°W, 800 m), leg., det.: O. Kroll	Co	13963	JQ973058
	Chile, Elqui River, Coquimbo (29.961°S, 71.322°W, 0 m), leg., det.: B. Werding	El	11077 11079	JQ973059 JQ973060
	Chile, Limari River, Ovalle (30.597°S, 71.176°W, 215 m), leg., det.: O. Kroll	Li	13965	JQ973061

*Heleobia* was sampled in the Altiplano and nearby sites during April–July 2007, October–December 2009, and January 2010. Specimens were collected in shallow waters by hand, in depths of up to 4 m by snorkeling and in depths of up to 38 m by boat using a small triangular dredge, and preserved in 70–80% ethanol. Snails were identified to species and subspecies based on original taxonomic descriptions and published keys (D’Orbigny 1835; Bavay 1904; Pilsbry 1924; Biese 1944, 1947; Haas 1955, 1957; Blume 1958; Dejoux 1992).

### DNA extraction, PCR amplification, and sequencing

Genomic DNA was obtained from individual specimens using the CTAB protocol described in Wilke et al. (2006). DNA vouchers were deposited at the University of Giessen Systematics and Biodiversity collection (UGSB) (see Table 1). Digital images of specimens were taken prior to consumptive DNA isolation and deposited in the UGSB database.

We obtained sequences of the mitochondrial cytochrome c oxidase subunit I (COI) gene with a target length of 658 base pairs (bps) (excluding 51-bp primer sequence). Forward and reverse primers for PCR amplification and DNA sequencing were LCO1490 (Folmer et al. 1994) and COR722b (Wilke and Davis 2000); the latter is based on primer HCO2198 (Folmer et al. 1994).

Bidirectional DNA sequencing was performed on a Long Read IR2 4200 sequencer (LI-COR, Lincoln, NE) using the Thermo Sequenase Fluorescent Labeled Primer Cycle Sequencing kit (Amersham Pharmacia Biotech, Piscataway, NJ). The protein-coding COI sequences, which are free of insertions and deletions in the Rissooidea (Wilke et al. 2001), were unambiguously aligned in BioEdit 7.0.4.1 (Hall 1999). The first base pairs behind the 3′ end of each primer were difficult to read. We therefore trimmed these regions, leaving a 638-bp-long overlapping fragment for the COI gene. New sequences were deposited in and additional sequences were taken from GenBank (see Table 1).

### Molecular dating: general problems and applicability in *Heleobia* spp

Molecular dating is a challenging task (e.g., Takahata 2007). At least four conditions have to be met for reliable molecular clock estimations (reviewed in Wilke et al. 2009): (1) the sampling design should be appropriate (i.e., ideally without missing lineages), (2) the gene(s) used should exhibit a low degree of rate heterogeneity within and among lineages, (3) the target gene should also have a good performance over the time frame of interest (i.e., a sufficient number of substitutions but no signs of significant substitutional saturation), and (4) robust internal calibration points and/or external molecular clock rates have to be available.

These conditions severely constrain the possibility of robust molecular dating. Although condition (1) may be satisfied assuming that the target taxa can be sampled, conditions (2) and (3) are violated by a substantial portion of genomic regions (e.g., Takahata 2007). Moreover, in the absence of robust internal calibration points, researchers frequently have to use external clock rates that are gene-specific and thus restrict the number of available genes. Consequently, most molecular dating studies use a single or very few genes, typically derived from mitochondrial DNA. Thus, the basis for molecular clock analyses differs from traditional phylogenetic investigations, which often include several genes derived from mitochondrial and nuclear DNA. Recent molecular clock analyses have incorporated sophisticated procedures for estimating the degree of rate heterogeneity, the error of the clock estimation, and the error of the external clock rate in order to partly compensate for this problem and to obtain meaningful time estimations. Efforts are also made to optimize external clock rates relative to their variability and specificity.

Here, we use such a “trait-specific” external clock rate (i.e., a rate that can be assigned to a range of taxa that share similar biological and life-history characteristics supposedly affecting rate heterogeneity) for the COI gene proposed by Wilke et al. (2009). This trait-specific rate has been shown to perform well in dioeceous aquatic protostomes from tropical and subtropical habitats that share a generation time of approximately one year and a body size of 2–50 mm. All of these conditions are met by *Heleobia*.

### Phylogenetic and molecular clock analyses

Fifty ingroup sequences and three cochliopid outgroup sequences (*Spurwinkia salsa*[GenBank accession number AF367633, Wilke et al. 2001], *Cochliopa* sp. [AF354762, Liu et al. 2001], and *Onobops jacksoni*[AF367645, Wilke et al. 2001]) were used for the phylogenetic and molecular clock analyses.

The best-fit model of sequence evolution was inferred based on the Bayesian information criterion by conducting dynamical likelihood ratio tests in jModelTest 0.1.1 (Posada 2008). Given that molecular clock analyses are particularly sensitive to substitution saturation (Wilke et al. 2009), we tested the degree of saturation using the entropy-based method of Xia et al. (2003) as implemented in DAMBE 5.2.9 (Xia and Lemey 2009). The input parameter for invariable sites was taken from jModelTest. The test did not indicate substantial saturation even under the conservative assumption of an asymmetrical tree as indicated by an “index of substitution saturation value” (Iss = 0.309) being significantly smaller than the respective critical value (Iss.c = 0.386).

We then tested whether the molecular clock hypothesis was accepted (i.e., whether a strict molecular clock can be assumed) using the Bayes factor (Kass and Raftery 1995) as model-choice criterion. In order to generate the Bayes factor, we conducted two Bayesian Inference analyses (no clock vs. strict clock assumption) in MrBayes 3.1.2 (Ronquist and Huelsenbeck 2003) with the nucleotide substitution model suggested by jModelTest (i.e., GTR + I + G). The individual analyses were terminated when the final average standard deviations of split frequencies in MrBayes reached values of <0.01. The posterior distributions were then used to estimate the Bayes factor. The harmonic means of −ln = 3142.4 and −ln = 3151.8 for the strict clock and no clock models, respectively, and the resulting Bayes factor of 18.8 provide strong evidence against the no clock model (see Kass and Raftery 1995 for an interpretation of Bayes factor values). Therefore, the strict clock model was used for subsequent phylogenetic analyses.

Priors for the Bayesian analysis were specified in BEAUti v.1.6.1 (Drummond and Rambaut 2007) as follows: site model = GTR + I + G (four gamma categories); clock model = strict clock (with a normal prior distribution as well as the COI trait specific clock rate of 0.017 and a standard deviation of 0.0034 suggested by Wilke et al. 2009). Phylogenetic reconstruction and estimation of the age of selected most recent common ancestors (MRCAs) were done in BEAST v.1.6.1 (Drummond and Rambaut 2007). We performed three independent analyses with different seeds and 10 Mio generations each.

During the runs, every 1000th tree was sampled and parameter convergence was monitored in Tracer v.1.5.0 (Drummond and Rambaut 2007). The combined set of trees showed both high ESS (effective sample size) values (>3000 for all major parameters) and a smooth frequency plot, indicating that the sampled trees well represent the posterior distribution. We then computed a consensus tree in TreeAnnotator v.1.6.1 (Drummond and Rambaut 2007) with the posterior probability limit set to zero and the first 10% of generations ignored as burn-in. From this tree, we obtained the means of selected time estimates and their 95% highest posterior density (HPD) intervals (i.e., the Bayesian analog to a confidence interval). This conservative error estimation incorporated both the errors of the phylogenetic analysis (i.e., the node-depth variation of individual Bayesian trees) and the error of the trait-specific COI clock rate. Note that we did not correct our clock estimates for ancestral polymorphism since the trait-specific Protostomia COI clock applied here is also uncorrected (Wilke et al. 2009). Given that this external rate is based on calibration points that have an average age of 3 My, there may be a small bias toward overestimation of divergence times for events younger than 3 My and underestimation for older events.

### Phylogeographical analysis

We constructed a parsimony haplotype network for Altiplano *Heleobia* utilizing TCS 1.21 (Clement et al. 2000), with the connection limit set to 95%.

## Results

### Phylogenetic analysis

The consensus Bayesian tree under the strict clock assumption is shown in Figure 2. This topology delineates four clades whose MRCAs predate the Pleistocene. These correspond to the genus *Heleobops* and the South American “*Heleobia*”*australis* (clade I), the European/western Asian genus *Semisalsa* (clade II), the genus *Heleobia* (clade III), and representatives of the Italian thermal water species “*Semisalsa*”*aponensis* (clade IV). The *Heleobia* clade (III) is subdivided into geographically distinct subclades. Clade IIIb is composed of taxa distributed in Altiplano and Andean localities to the west and north of this plateau (herein referred to as the “northern *Heleobia* clade”); clade IIIa is composed of species distributed slightly to the south of the Altiplano (“southern *Heleobia* clade”). Although many of the young clades are not well supported, suggesting a need for additional phylogeographical analyses (see below), all of the deeper nodes pertinent to the goals of this study are supported by Bayesian posterior probability (BPP) values of ≥0.95. These are the MRCAs of the Semisalsinae (split A in Fig. 2), the northern *Heleobia* clade (spilt B), and the Altiplano taxa (split C).

Our results indicate that the Lake Titicaca *Heleobia* assemblage is not monophyletic but instead forms a clade together with other Altiplano species and two species that are distributed in areas adjacent to the Altiplano (i.e., Loa River and Lake Langui Layo). The sister to this clade is a congener distributed to the north of the Altiplano (*Heleobia* cf. *cumingii*, Urubamba River).

### Molecular clock analysis

We only estimated the age of those clades that are well supported and pertinent to the goals of this paper. The MRCA of Semisalsinae exemplars is 3.57 My old with a 95% HDP interval of 2.14–5.43 My (see split A). The MRCA of the Altiplano fauna and its extralimital sister group (i.e., the northern *Heleobia* clade) is 0.92 (0.46−1.52) My old (split B) and the MRCA of the Altiplano fauna is only 0.53 (0.28–0.80) My old (split C).

### Network analysis

**Figure 3:**
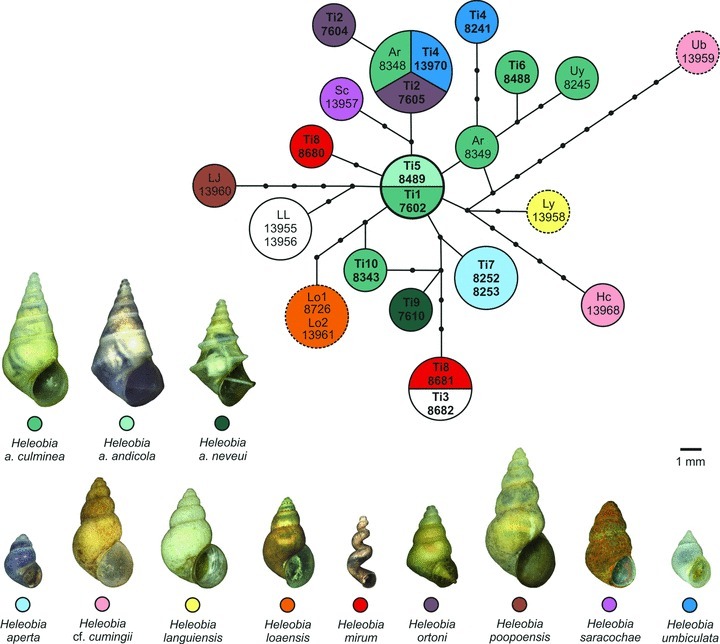
Statistical parsimony haplotype network for members of the northern *Heleobia* clade. Haplotypes are color-coded by nominal taxa (white circles indicate specimens that either could not be identified or that appear to be transitional forms). Circle areas are scaled in proportion to the number of specimens sharing the respective haplotype. Missing haplotypes are indicated by black dots. Haplotypes from Lake Titicaca and from areas outside the Altiplano are marked by bold face and dashed circles, respectively. The haplotype with the highest probability of being ancestral is indicated by a bold circle. Specimens are labeled with locality codes and DNA voucher numbers (see Table 1).

The COI-based TCS network for the northern *Heleobia* clade (corresponding to clade IIIb in Fig. 2) is shown in Figure 3. It consists of 19 haplotypes, six of which are shared. The haplotype with the highest probability of being ancestral is shared by two specimens from Lake Titicaca (Ti5 8489 and Ti1 7602). Specimens from Lake Titicaca and other Altiplano sites cluster together as in the Bayesian tree. Extralimital specimens (i.e., Ly 13958, Ub 13959, and Lo1 8726/Lo2 13961) have terminally positioned (young) haplotypes.

Haplotypes derived from the same species do not always cluster together and substitutional differences among species are generally low. Furthermore, some haplotypes are shared among species. This implies incomplete lineage sorting, hybridization, or confused taxonomy. Note that some specimens were morphologically transitional between species (indicated as white circles in Fig. 3), further suggesting hybridization.

## Discussion

### Lake Titicaca: reservoir versus cradle function

The generation of endemic diversity in ancient lakes has been generally attributed to multiple independent colonization events (reservoir function) or intralacustrine diversification of single lineages (cradle function). Previous studies typically favored the reservoir function model (e.g., Rossiter and Kawanabe 2000) or a combination of both models (Wilson et al. 2004). Most of the recently published data, however, support the intralacustrine speciation model. Examples include faunas of Lake Baikal (e.g., Kaygorodova et al. 2007), Lake Tanganyika (e.g., Marijnissen et al. 2006), Lake Malawi (e.g., Schultheiß et al. 2009, 2011), and Lake Ohrid (e.g., Albrecht et al. 2006; Wilke et al. 2007; Wysocka et al. 2008; Trajanovski et al. 2010). Our findings are highly pertinent to this subject and suggest the following:

1. The Altiplano *Heleobia* fauna (together with several species distributed in close proximity to this region) is a species flock.
2. The evolutionary development of this flock postdates the age of the lake, suggesting that the flock is a product of intralacustrine and/or other local speciation events.
3. The haplotype with the highest probability of being ancestral is shared by two Lake Titicaca specimens (Fig. 3). Furthermore, all but one internal haplotype in this network are from Lake Titicaca specimens (the only exception is haplotype Ar 8349 from Lake Arapa, a small satellite lake only 7 km north of Lake Titicaca). This suggests that Lake Titicaca was the principal site of speciation within the region (although not necessary the center of origin of the species flock).

Our finding of primarily intralacustrine speciation is not contradicted by the distribution of a few members of the flock in extralimital areas closely proximal to the Altiplano. The haplotypes of these taxa are peripherally positioned in the phylogeographical network (Fig. 3) and thus are possibly derived from Altiplano taxa. These extralimital areas may have been colonized via drainages originating close to the Altiplano such as the Loa (*Heleobia loaensis*), the Urubamba (*H.* cf. *cumingii*), and the Jilatunga and Quelcapampa rivers. The latter two drain into Lake Langui Layo, which contains endemic *H. languiensis*. However, given that some of these haplotypes cluster as outgroups to Altiplano/Lake Titicaca haplotypes in the phylogenetic analysis (Fig. 2), we cannot exclude the possibility that the Altiplano was colonized by an MRCA originating in these areas.

### Correlation between paleohydrology and molecular evolution

One of our most striking results of this study is the close correlation of cladogenetic events with prior hydrological configurations of Lake Titicaca. The age of the Cabana episode approximates the onset of diversification of the *Heleobia* flock (event B in Fig. 2). Although this episode may have separated ancestral Altiplano taxa from those distributed to the north of the plateau, the tree topology does not indicate an increased rate of speciation at this time. Substantial diversification occurred coincident with the Ballivián episode (event C in Fig. 2), giving rise to all extant Altiplano taxa. Note that the 95% HDP intervals of splits B and C extend beyond the duration of the respective “paleolakes” (which, in turn, are also poorly dated). Therefore, a random correlation cannot be ruled out completely. However, the pattern observed for the *Heleobia* flock matches that found in another group of regionally endemic species, the “Agassii” pupfish complex (genus *Orestias*). Based on a different genetic marker, Lüssen (2003) inferred that lineage diversification within this complex occurred 0.53–0.88 My ago. This well conforms to the 95% HDP interval of 0.28–0.80 My that we estimated for the diversification of the *Heleobia* flock. Lüssen (2003) similarly associated colonization of the central and southern Altiplano by pupfishes with the Ballivián episode.

### Spatial levels of endemicity

Ancient lakes are typically characterized by a relative large number of endemic species. There is also a low level of faunal exchange between the lake and neighboring water bodies; endemic lacustrine organisms typically outcompete invading species but are often inferior outside the lake system (a pattern first noted by Brooks 1950 and today sometimes referred to as eco-insularity, Albrecht and Wilke 2008; Wilke et al. 2010).

The spatial scale of these patterns varies. Endemism and eco-insularity have been documented in isolated areas in a lake (point endemism), at the lake level, at the level of the lake and associated water bodies, and at the entire lake basin level (e.g. Albrecht and Wilke 2008; Schreiber et al. 2012).

There have been no comprehensive assessments of the processes that shape these spatial patterns. Although the watersheds of ancient lake have been little investigated biologically, there is some evidence suggesting that in relatively stable lacustrine systems, endemism is mostly concentrated at the lake level or even within the entire (see Shirokaya 2007 for Lake Baikal, Michel et al. 2004 for Lake Tanganyika, and Albrecht and Wilke 2008 for Lake Ohrid). In less-stable ancient lake systems, there often is evidence of increased faunal exchange within the entire watershed, thus leading to a spatial expansion of endemism (see Genner et al. 2007 and Schultheiß et al. 2009, 2011 for Lake Malawi; Albrecht et al. 2012 for Lake Prespa; and Dumont 1998 for the Caspian Sea).

The *Heleobia* flock conforms to the latter pattern. The network analysis (Fig. 3) implies that Lake Titicaca is the center of diversification in the Altiplano region, which is, in turn, the center of diversification of the northern *Heleobia* clade. However, this hierarchy of contributing biogeographic processes has been somewhat confused by recent immigration and/or emigration events of riverine taxa, resulting in a contemporary pattern that is largely nonhierarchical—that is, there is a single genetic break between the northern and southern *Heleobia* clades.

Our study also provides a possible explanation for why the total number of endemic species in Lake Titicaca, in general, and the number of species flocks, in particular, is low compared to other ancient lake faunas. The biological attributes of the regional species flocks (i.e., *Orestias*, *Hyalella*, *Heleobia* flocks) suggest that their ancestors all had the potential for adapting to a brackish water lifestyle. *Heleobia* is a primarily brackish water group (Hershler and Thompson 1992), *Hyalella* contains widespread brackish water representatives outside the Altiplano (Väinölä et al. 2008), and the potential sister groups of *Orestias* (i.e., the pupfish genera *Cyprinodon* and *Aphanius*) contain species living in estuarine or otherwise highly mineralized waters (Lüssen et al. 2003). This suggests that the groups that diversified in the Altiplano have a relatively high salinity tolerance and their progenitors may have been able to tolerate severe changes in salinity during Quaternary paleohydrologic episodes. In contrast, taxa having a lower salinity tolerance may have been prone to extinction (e.g., Benavides 2005) or possibly colonized the Altiplano only within the past 3600 years after the lakes returned to freshwater conditions (Ybert 1992).

### Concluding remarks

Our sampling design and choice of marker were optimized to address evolutionary questions above the species level and to utilize a reliable trait-specific clock rate for molecular dating. Future studies should focus on sampling more populations. Moreover, the identification of potential fossil calibration points (e.g., earliest appearance of fossil *Heleobia* in the Altiplano) would assist molecular dating by enabling the use of multilocus approaches.

The species-level taxonomy of Titicaca/Altiplano *Heleobia* needs to be better resolved. Our results imply incomplete lineage sorting and hybridization as previously observed in regional pupfishes. An analysis of highly variable nuclear markers (such as microsatellites) combined with morphological and ecological studies may be useful in this regard.

Nonetheless, we suggest that adding more markers would not change the results or conclusions derived from this paper because we used a highly conservative molecular dating approach that took into consideration the node-depth variation of the COI trees.

Our findings contribute to a rapidly growing body of evidence suggesting that (1) the high degree of endemic biodiversity in ancient lakes is largely the product of intralacustrine speciation, (2) much of this diversification is geologically recent and was triggered by lake-level and associated environmental changes, and (3) the spatial scale of endemism is correlated with watershed stability.

## References

[b2] Albrecht C, Wilke T (2008). Ancient Lake Ohrid: biodiversity and evolution. Hydrobiologia.

[b1] Albrecht C, Trajanovski S, Kuhn K, Streit B, Wilke T (2006). Rapid evolution of an ancient lake species flock: freshwater limpets (Gastropoda: Ancylidae) in the Balkan lake Ohrid. Org. Divers. Evol.

[b3] Albrecht C, Hauffe T, Schreiber K, Wilke T (2012). Assessing mollusc biodiversity in an endangered European ancient lake system: lakes Prespa and Mikri Prespa in the Balkans. Hydrobiologia.

[b4] Allmendinger RW, Jordan TE, Kay SM, Isacks BI (1997). The evolution of the Altiplano-Puna plateau of the Central Andes. Annu. Rev. Earth Planet. Sci.

[b5] Argollo J, Mourguiart P (2000). Late Quaternary climate history of the Bolivian Altiplano. Quat. Int.

[b7] Baker PA, Fritz SC, Garland J, Ekdahl E (2005). Holocene hydrologic variation at Lake Titicaca, Bolivia/Peru, and its relationship to North Atlantic climate variation. J. Quat. Sci.

[b8] Bavay A (1904). Mission de Crequi-Montfort et Senechal de la Grange en Amerique du Sud. Mollusques terrestres et fluviatilis recoltes par le Dr Neveu-Lemaire. Bull. Soc. Zool. France.

[b9] Benavides E (2005). The *Telmatobius* species complex in Lake Titicaca: applying phylogeographic and coalescent approaches to evolutionary studies of highly polymorphic Andean frogs. Monogr. Herpetol.

[b10] Betancourt JL, Latorre C, Rech JA, Quade K, Rylander A (2000). A 22,000-year record of monsoonal precipitation from Northern Chile's Atacama Desert. Science.

[b11] Biese WA (1944). Revisión de los moluscos terrestres y de agua dulce provistos de concha de Chile. Parte I, Familia Amnicolidae. Boletín del Museo de Historia Natural, Chile.

[b12] Biese WA (1947). Revisión de los moluscos terrestres y de agua dulce provistos de concha de Chile. Parte I, Familia Amnicolidae (continuación). Boletín del Museo de Historia Natural, Chile.

[b13] Blard P-H, Sylvestre F, Tripati AK, Claude C, Causse C, Coudrain A, Condom T, Seidel J-L, Vimeux F, Moreau C (2011). Lake highstands on the Altiplano (Tropical Andes) contemporaneous with Heinrich 1 and the Younger Dryas: new insights from 14 C, U-Th dating and δ18 O of carbonates. Quat. Sci. Rev.

[b14] Blume W (1958). Littoridinen aus dem Titicacasee (Mollusca). Opuscula Zoologica.

[b15] Brooks JL (1950). Speciation in ancient lakes. Quat. Rev. Biol.

[b17] Clement M, Posada D, Crandall KA (2000). TCS: a computer program to estimate gene genealogies. Mol. Ecol.

[b18] Cross SL, Baker PA, Seltzer GO, Fritz SC, Dunbar RB (2000). A new estimate of the Holocene low-stand level of Lake Titicaca (16°S) and implications for regional paleohydrology. Holocene.

[b19] Dejoux C, Dejoux C, Ilties A (1992). The Mollusca. Lake Titicaca—a synthesis of limnological knowledge.

[b20] Dejoux C (1994). Lake Titicaca. Archiv für Hydrobiologie—Adv. Limnol.

[b37] de Lattin G (1967). Grundriss der Zoogeographie.

[b21] D’Orbigny A (1835). Synopsis terrestrium et fluviatilium Molluscorum in suo per American meridionalem itinere collectorum. Mag. Zool.

[b22] Drummond AJ, Rambaut A (2007). BEAST: Bayesian evolutionary analysis by sampling trees. BMC Evol. Biol.

[b23] Dumont HJ (1998). The Caspian Lake: history, biota, structure, and function. Limnol. Oceanogr.

[b24] Falniowski F, Szarowska M, Sirbu I, Hillebrand A, Baciu M (2008). *Heleobia dobrogica* (Grossu & Negrea, 1989) (Gastropoda: Rissooidea: Cochliopidae) and the estimated time of its isolation in a continental analogue of hydrothermal vents. Molluscan Res.

[b25] Folmer O, Black M, Hoeh W, Lutz RA, Vrijenhoek RC (1994). DNA primers for amplification of mitochondrial cytochrome c oxidase subunit I from diverse metazoan invertebrates, Mol. Mar. Biol. Biotechnol.

[b26] Fornari M, Risacher F, Féraud G (2001). Dating of paleolakes in the central Altiplano of Bolivia. Palaeogeogr. Palaeoclimatol. Palaeoecol.

[b27] Fritz SC, Baker PA, Seltzer GO, Ballantyne A, Tapia PM, Cheng H, Edwards RL (2007). Quaternary glaciations and hydrologic variation in the South American tropics as reconstructed from the Lake Titicaca drilling project. Quat. Res.

[b28] Genner MJ, Nichols P, Carvalho GR, Robinson RL, Shaw PW, Smith A, Turner GF (2007). Evolution of a cichlid fish in a Lake Malawi satellite lake. Proc. R. Soc. B Biol. Sci.

[b29] González ER, Watling L (2003). A new species of *Hyalella* from the Patagonia, Chile with re-description of *H. simplex* Schellenberg, 1943 (Crustacea: Amphipoda). J. Nat. Hist.

[b30] Haas F (1955). The Percy Sladen Trust Expedition to Lake Titicaca in 1937, 17. Mollusca: Gastropoda. Transactions of the Linnean Society of London.

[b31] Haas F (1957). Eine neue endemische Schnecke aus dem Titikaka-See. Arch. Moll.

[b32] Hall TA (1999). BioEdit: a user-friendly biological sequence alignment editor and analysis program for Windows 95/98/NT. Nucl. Acids Symp. Ser.

[b33] Hershler R, Thompson FG (1992). A review of the aquatic gastropod subfamily Cochliopinae (Prosobranchia: Hydrobiidae). Malacol. Rev. Suppl.

[b34] Hershler R, Liu HP, Mulvey M (1999). Phylogenetic relationships within the aquatic snail genus *Tryonia*: implications for biogeography of the North American Southwest. Mol. Phylogenet. Evol.

[b35] Kass RE, Raftery AE (1995). Bayes factors. J. Am. Stat. Assoc.

[b36] Kaygorodova IA, Sherbakov DYu, Martin P (2007). Molecular phylogeny of Baikalian Lumbriculidae (Oligochaeta): Evidence for recent explosive speciation. Comp. Cytogenet.

[b38] Lavenú A (1981). Origine et évolution néotecto-nique du lac Titicaca. Rev. Hydrobiol. Trop.

[b39] Lavenú A, Fornari M, Sebrier M (1984). Existence de deux nouveaux épisodes lacustres Quaternaires dans l'Altiplano Péruvo-Bolivien. Cah. O.R.S.T.O.M., ser. Geol.

[b40] Lavenú A, Dejoux C, Ilties A (1992). Formation and geological evolution. Lake Titicaca—a synthesis of limnological knowledge.

[b41] Lavenú A (1995). Geodinamica Plio-Cuaternaria en los Andes Centrales: El Altiplano Norte de Bolivia. Revista Tecnica de YPFB.

[b42] Liu H-P, Hershler R, Thompson FG (2001). Phylogenetic relationships of the Cochliopinae (Rissooidea: Hydrobiidae): an enigmatic group of aquatic gastropods. Mol. Phylogenet. Evol.

[b43] Lüssen A (2003). Zur Systematik, Phylogenie und Biogeographie chilenischer Arten der Gattung *Orestias* Valenciennes, 1839 (Teleostei, Cyprinodontidae): morphologische, biochemische und molekularbiologische Befunde.

[b44] Lüssen A, Falk TM, Villwock W (2003). Phylogenetic patterns in populations of Chilean species of the genus *Orestias* (Teleostei: Cyprinodontidae): results of mitochondrial DNA analysis. Mol. Phylogenet. Evol.

[b45] Marijnissen SAE, Michel E, Daniels SR, Erpenbeck D, Menken SBJ, Schram FR (2006). Molecular evidence for recent divergence of Lake Tanganyika endemic crabs (Decapoda: Platythelphusidae). Mol. Phylogenet. Evol.

[b46] Marshall LG, Sempere T, Suarez-Soruco R (1991). The Eocene to Pleistocene vertebrates of Bolivia and their stratigraphie context: a review. Fosiles y facies de Bolivia: Vertebrados.

[b47] Martens K (1997). Speciation in ancient lakes (review). Trends Ecol. Evol.

[b48] Michel E, Todd JA, Cleary DFR, Kingma I, Cohen SA, Genner MJ (2004). Scales of endemism: challenges for conservation and incentives for evolutionary studies in a gastropod species flock from Lake Tanganyika. J. Conchol.

[b49] Pawley A, Fritz SC, Baker PA, Seltzer GO, Dunbar R, Munawar M, Hecky R (2001). The biological, chemical, and physical limnology of Lake Titicaca, Bolivia/Peru. The Great Lakes of the World; food web, health and integrity. Ecovision world monograph series.

[b50] Pilsbry HA (1924). South American land and freshwater molluscs. Notes and descriptions. Proc. Acad. Nat. Sci. Phila.

[b51] Posada D (2008). jModelTest: phylogenetic model averaging. Mol. Biol. Evol.

[b52] Ronquist F, Huelsenbeck JP (2003). MrBayes 3: Bayesian phylogenetic inference under mixed models. Bioinformatics.

[b53] Rossiter A, Kawanabe H (2000). Ancient lakes: biodiversity, ecology and evolution.

[b54] Schreiber K, Hauffe T, Albrecht C, Wilke T (2012). The role of barriers and gradients in differentiation processes of pyrgulinid microgastropods of Lake Ohrid. Hydrobiologia.

[b55] Schultheiß R, Van Bocxlaer B, Wilke T, Albrecht C (2009). Old fossils–young species: evolutionary history of an endemic gastropod assemblage in Lake Malawi. Proc. R. Soc. Lond. B.

[b56] Schultheiß R, Wilke T, Jørgensen A, Albrecht C (2011). The birth of an endemic species flock: demographic history of the *Bellamya* group (Gastropoda, Viviparidae) in Lake Malawi. Biol. J. Linn. Soc.

[b57] Shirokaya A (2007). A new species of *Gerstfeldtiancylus* Starobogatov, 1989 (Pulmonata: Basommatophora: Acroloxidae) from Lake Baikal. Zootaxa.

[b58] Takahata N (2007). Molecular clock: an anti-neo-Darwinian legacy. Genetics.

[b59] Trajanovski S, Albrecht C, Schreiber K, Schultheiß R, Stadler T, Benke M, Wilke T (2010). Testing the spatial and temporal framework of speciation in an ancient lake species flock: the leech genus *Dina* (Hirudinea: Erpobdellidae) in Lake Ohrid. Biogeosciences.

[b60] Väinölä R, Witt JDS, Grabowski M, Bradbury JH, Jazdzewski K, Sket B (2008). Global diversity of amphipods (Amphipoda; Crustacea) in freshwater. Hydrobiologia.

[b62] Wilke T, Davis GM (2000). Infraspecific mitochondrial sequence diversity in *Hydrobia ulvae* and *Hydrobia ventrosa* (Hydrobiidae: Rissooidea: Gastropoda): do their different life histories affect biogeographic patterns and gene flow?. Biol. J. Linn. Soc.

[b61] Wilke T, Davis GM, Falniowski A, Giusti F, Bodon M, Szarowska M (2001). Molecular systematics of Hydrobiidae (Mollusca: Gastropoda: Rissooidea): testing monophyly and phylogenetic relationships. Proc. Acad. Nat. Sci. Phila.

[b63] Wilke T, Davis GM, Qiu D, Spear RC (2006). Extreme mitochondrial sequence diversity in the intermediate schistosomiasis host *Oncomelania hupensis robertsoni*: another case of ancestral polymorphism?. Malacologia.

[b64] Wilke T, Albrecht C, Anistratenko VV, Sahin SK, Yildirim MZ (2007). Testing biogeographical hypotheses in space and time: faunal relationships of the putative ancient Lake Egirdir in Asia Minor. J. Biogeogr.

[b65] Wilke T, Schultheiß R, Albrecht C (2009). As time goes by: a simple fool's guide to molecular clock approaches in invertebrates. Am. Malac. Bull.

[b66] Wilke T, Schultheiß R, Albrecht C, Bornmann N, Trajanovski S, Kevrekidis T (2010). Native *Dreissena* freshwater mussels in the Balkans: in and out of ancient lakes. Biogeosciences.

[b67] Wilson AB, Glaubrecht M, Meyer A (2004). Ancient lakes as evolutionary reservoirs. Evidence from the thalassoid gastropods of Lake Tanganyika. Proc. R. Soc. Lond.

[b69] Wirrmann D, Dejoux C, Ilties A (1992). Morphology and bathymetry. Lake Titicaca—a synthesis of limnological knowledge.

[b68] Wirrmann D, Ybert J-P, Mourguiart P, Dejoux C, Ilties A (1991). A 20,000 years paleohydrological record from Lake Titicaca. Lake Titicaca—a synthesis of limnological knowledge.

[b70] Wysocka A, Kostoski G, Kilikowska A, Wróbel B, Sell J (2008). The *Proasellus* (Crustacea, Isopoda) species group, endemic to the Balkan Lake Ohrid: a case of ecological diversification?. Fundam. Appl. Limnol.

[b72] Xia X, Lemey P, Lemey P, Salemi M, Vandamme A-M (2009). Assessing substitution saturation with DAMBE. The Phylogenetic Handbook: a practical approach to DNA and protein phylogeny.

[b71] Xia X, Xie Z, Salemi M, Chen L, Wang Y (2003). An index of substitution saturation and its application. Mol. Phylogenet. Evol.

[b73] Ybert J-P, Dejoux C, Ilties A (1992). Ancient lake environment as deduced from pollen analysis. Lake Titicaca—a synthesis of limnological knowledge.

